# Articulatory effects on perceptions of men’s status and attractiveness

**DOI:** 10.1038/s41598-023-29173-z

**Published:** 2023-02-14

**Authors:** Sethu Karthikeyan, David A. Puts, Toe Aung, Jennifer K. Link, Kevin Rosenfield, Alexander Mackiel, Allisen Casey, Kaelyn Marks, Michele Cristo, Jenny Patel, Aliza Santos, Glenn Geher

**Affiliations:** 1 Communication Sciences and Disorders Department, Pace University, New York, NY 10038 USA; 2 Department of Anthropology, The Pennsylvania State University, University Park, PA 16802 USA; 3 Psychology and Counseling Department, Immaculata University, Immaculata, PA 19345 USA; 4 Animal Behavior Graduate Group, University of California, Davis, CA 95616 USA; 5 Department of Psychology, State University of New York, New Paltz, NY 12561 USA

**Keywords:** Evolution, Psychology

## Abstract

Research on heterosexual mating has demonstrated that acoustic parameters (e.g., pitch) of men’s voices influence their attractiveness to women and appearance of status and formidability to other men. However, little is known about how men’s tendency to clearly articulate their speech influences these important social perceptions. In the current study, we used a repeated-measures design to investigate how men’s articulatory clarity or conformity influenced women’s (N = 45) evaluations of men’s attractiveness for both short- and long-term relationships, and men's (N = 46) evaluations of physical formidability and prestige. Results largely supported our hypotheses: men who enunciated phonemes more distinctly were more attractive to women for long-term relationships than short-term relationships and were perceived by other men to have higher prestige than physical dominance. These findings suggest that aspects of articulatory behavior that influence perceptions of prestige and long-term mating attractiveness may indicate an early social history characterized by high socioeconomic status, likely owing to crystallization of articulatory patterns during the critical period of language development. These articulatory patterns may also be honest signals of condition or disposition owing to the nature of complex, multicomponent traits, which deserve further empirical attention.

## Introduction

We shape our vocal tracts using articulatory organs such as the tongue, lips, teeth, jaw, and vocal folds to produce sounds that have no inherent meaning but rather acquire meanings and conventional styles of articulation through communities of speakers. In a given language, speech sounds are frequently allophonic—acceptable phonetic realizations that do not alter word meaning. Nevertheless, allophones may convey different forms of social information. In American English, there are two allophones for the word-final stop /t/ that are in free variation, i.e., not determined by the phonetic environment. For example, the word “boat” may be produced with the aspirated /t/, i.e., with a perceptible release of air as with the /t/ in “tea”, or with the unreleased /t/, i.e., without releasing air, where “boat” may sound more like “bow” in “rainbow,” usually coupled with a glottal closure, i.e., closing the space between the vocal folds, producing a glottal stop^1^. Although it is not necessarily associated with a particular region^2–5^, there is consensus across the different varieties of English that the glottal variant has been historically associated with working-class speech. Stigma associated with the glottal form has reduced over time, with even speakers of Received Pronunciation (RP) employing it, but the glottal form has not attained the prestige accorded to the clearly enunciated /t/ ^6^.

Some studies report that females, compared to males, produce the more clearly enunciated /t/, with longer time intervals between the release of the /t/ and the onset of vocal fold vibration or voicing (voice onset time or VOT) for the following vowel ^7–9^. With respect to the use of the glottal variant, whereas some studies report more women than men glottalizing word final /t/’s^3^ others show fewer men than women releasing their /t/’s ^10^(see ^11^ for effects in the opposite direction). Further, reduction in phonetic forms, in general, such as “in” for “ing” (e.g., “writin” vs. “writing”) and shorter vowel spaces or less distinctive vowel sounds have been documented more frequently in men than in women ^10,12^. These forms contrast with expectations of “standard speech” (e.g., RP), and other “standard” varieties influenced by RP, which generally conveys the impression of deliberate and careful articulation rendering the speakers’ regions of origin unpredictable^13^. /t/ articulation, in particular, has been linked to articulateness or learnedness^11,14–16^.

These observations also align with earlier proposals depicting a sense of covert prestige among male speakers of non-standard varieties of English^17^. The working-class speech forms carry connotations of independence and authority leading to attributions of masculinity^18,19^, as evidenced in the choice of non-standard linguistic varieties for masculine movie roles^20^, misjudgments of girls as boys based on their enhanced use of non-standard linguistic forms^21^, these forms constituting a greater proportion of child-directed speech to boys^22^, and the underreporting and overreporting of the use of standard forms by men and women, respectively^23^.

Although developmental, acoustic, sociolinguistic, and clinical literatures shed light on how speech articulation is linked to social perceptions and roles, little attention has been directed toward the topic from an evolutionary perspective. An evolutionarily informed approach can provide a useful theoretical framework that integrates seemingly disparate findings and suggests new hypotheses. Sexual selection theory may be particularly illuminating. Sexual selection favors traits that aid in competition for mates and has been studied in many species across a diversity of taxa, including primates^24^. Darwin^25^ proposed sexual selection to explain extravagant traits such as peacocks’ tails and deer stags’ antlers that appeared to impede survival. Such traits can compensate for their survival and energetic costs by increasing males’ aesthetic appeal to females and conferring an advantage in agonistic interactions with other males. He speculated, based on the general plan of vocal structure and function across species, that courtship displays and challenges to rivals in contests must have facilitated the earliest phase of vocal-verbal innovation in humans^25–27^.

A growing literature supports the hypothesis that sexual selection played important roles in shaping human vocal-verbal parameters. For example, men’s vocal pitch (perceptual correlate of fundamental frequency, *f*_o_) is negatively correlated with testosterone levels ^28–30^ and body size ^29,31–35^, is heritable^36^, and may predict immune function^37,38^. Deep voiced men are also perceived as larger, stronger, and better fighters^39–44^, indicating that vocal pitch may be an honest indicator of condition and physical formidability. Further, vocal pitch varies according to social context^45,46^, including the perceived qualities of interlocutors^41^; men who perceived their interlocutors as higher in relative physical dominance or prestige raised their vocal pitch in conversation^41,47,48^, and deep voiced men are perceived to have greater aggressive intent^49^, suggesting that variability in the use of vocal pitch renders it an honest signal of relative formidability in particular social contexts.

Low voice pitch in men systematically predicts perceptions of attractiveness, number of sexual partners, and offspring^50–53^. Women have been found to prefer deep masculine voices^40^, particularly when evaluating male voices for short-term sexual relationships^51,54^. However, women may prefer a raised male voice pitch in contexts where they prioritize long-term investment and commitment from males, for example, when breast feeding^52,55,56^.

Articulatory behavior likewise exhibits several features of traits that may have been shaped by sexual selection, such as sexual dimorphism, variability, and association with components of mating success^57–60^. For example, evidence reviewed above indicates that articulatory clarity is sexually differentiated, and other findings indicate associations with gonadal hormone concentrations in adults ^9,61,62^. The clarity with which American men articulated the “t” sound in word-final positions correlated with circulating testosterone^62^. Moreover, there are individual differences in articulatory clarity^63–65^, and articulatory clarity measured through consonant accuracy in conversational speech and repetition tasks has been found to be moderately heritable^66,67^. Enhanced clarity of women’s speech predicted attractiveness evaluations^12^, and women perceived the accent accorded low sociolinguistic status as low in socioeconomic status, e.g., “runnin” vs. “running,” and “wridden” vs. “written”^68^.

Of interest is the fact that laryngeal manipulation interacts with both vocal pitch and articulation. Laryngeal lowering and raising decreases and raises pitch^69^. Voiceless (i.e., absent vocal fold vibration) aspirated consonants such as /t/ are facilitated by a high laryngeal position^70–72^; by contrast, a lower laryngeal position supports not only voiced consonants such as /d/ (i.e., stop consonants with vocal fold vibration occurring at about the same time as the release of the oral gesture^70,73–75^), but also lax (weak) consonantal production^72^. This is relevant because the aspirated /t/ is sometimes supplanted by a weak version (e.g., “wridden” for “written”^1^). An alternative replacement for the aspirated /t/ is the glottal variant. Glottalization, in general, is characterized by low vocal pitch^76,77^, sometimes referred to as creaky phonation^78,79^. Thus, the two allophonic variants of the aspirated /t/, the glottal variant and the tap, may be facilitated by a low laryngeal position.

It is possible that men with varying levels of circulating testosterone vary in the tendencies to move their larynges up or down influencing not only pitch but also articulatory patterns. Laryngeal raising also shortens the vocal tract raising formant frequencies; as formants track body size, raised formants serve as a cue for smaller size. For example, mothers engaging in baby talk with their infants raise their larynges, presumably to signal a non-threatening smaller persona, which has the unintended consequence of enhancing speech clarity by expanding the vowel space^80^.

Laryngeal lowering has the opposite effect of signaling dominance through not only elongating the vocal tract and lowering formants, but also lowering pitch and aiding consonant weakening or glottalization. If testosterone variations track individual differences in vocal pitch, formants, and the frequency of word-final /t/ aspiration, then it is reasonable to hypothesize that American men may be adopting one of two strategies, men with higher levels of testosterone favoring a relatively constant lowered larynx strategy, and others with lower levels of testosterone raising it momentarily and being faithful to the distinctive feature of the sound, namely the /t/ release with aspiration. Although the speakers adopting the unreleased /t/ strategy may be perceived as higher in physical dominance, which is potentially a downstream effect of maintaining a low larynx position, the aspirated /t/ speakers may be perceived as higher in prestige as they momentarily raise their larynges to be faithful to phonology. Prestige is freely bestowed to individuals who display skills or knowledge that are deemed useful by the community, as opposed to status via physical dominance, which is attained through coercion and threat of force^81,82^. Also, note that testosterone has shown an inverse relationship with prestige^83^. We therefore hypothesized that sexual selection may have shaped neuropsychological factors underlying male articulatory patterns. From this hypothesis, following the hierarchical theoretical structure of evolutionary psychology^84^, we generated the following predictions on evaluations of attractiveness by women and evaluations of status by men:

1. The unreleased /t/ will receive higher short-term mating attractiveness scores than (a) long-term mating attractiveness scores, and (b) those received by the aspirated /t/.
2. The aspirated /t/ will receive higher long-term mating attractiveness scores than (a) short-term mating attractiveness scores, and (b) those received by the unreleased /t/.
3. The unreleased /t/ will receive higher physical dominance scores than (a) prestige scores, and (b) those received by the aspirated /t/.
4. The aspirated /t/ will receive higher prestige scores than (a) physical dominance scores, and (b) those received by the unreleased /t/.

An additional aspect that affects mating-relevant evaluations is perceived similarity to potential mates. Given that (a) the degree of perceived similarity to potential mates may influence attractiveness ratings^85,86^, (b) pronunciation differences are a dedicated feature of social categorization^87^, and (c) native speakers of American English can accurately sort speakers into different regional dialect groups based on listening to brief speech samples^88^, we hypothesized that higher perceived similarity will lead to higher attractiveness ratings.

## Method

### Participants

The study was approved by the Human Research and Ethics Board at the State University of New York. All methods were performed in accordance with relevant guidelines and regulations. Participants were recruited through the Qualtrics Panel service, and mass email lists/SONA systems. Psychology students who participated were offered subject pool credit. Forty-five self-reported heterosexual females (mean age 20.51 years; range 18–24) and 46 self-reported heterosexual males (mean age 23.3 years; range 18–59) participated. All participants reported being native speakers of American English and generally healthy with no speech and hearing issues.

Sixty percent of the females identified as white; 17% as African American, 13% as Asian American and 7% as non-white Latino. Participants met inclusion criteria of reporting not currently taking hormonal contraception. All reported being native speakers of American English. Participants reported that they were from the northeastern (51%), southwestern (24%), midwestern (13%), or western (11%) United States.

Fifty-two percent of male participants identified as white, 20% as African American, 17% as Asian American, 4% as Hispanic, 4% as non-white Latino, and 2% as Latino. Participants reported that they were from the northeastern (52%), southwestern (24%), midwestern (13%), or western (11%) United States.

### Materials

The two parts of the current study—attractiveness evaluations made by female participants and social status evaluations made by male participants—used speech samples collected by Kempe et al.^62^. A single speech sample from the data set consisted of the following series of 15 words.

*beat, bit, bet, bait, bat, but, bout, bye, book, boot, boat, bought, bird, car, and ago*

Speech samples from 80 self-reported white, heterosexual males (ages 18–26 years) originating mostly from the midwestern U.S. were used as stimuli for ratings. Samples were selected so that half (40 of 80) included an aspirated /t/ in all 10 of the /t/-final words, and the remainder never included an aspirated (unreleased) final /t/. Words without a final /t/ were retained in speech stimuli so as not to draw participants’ attention to the target sound. Acoustic parameters for mean *f*_o_, formant position (the average standardized formant value for the first four formants), and jitter (cycle-to-cycle variations in *f*_o_) were extracted from the speech samples using Praat software^89^ (Version 6.1.53) and script that is publicly available on the OSF^90^.

The study was conducted online via Qualtrics survey software. Two versions consisting of different sets of 20 aspirated /t/s and 20 unreleased /t/s were created for both attractiveness evaluations and status evaluations to reduce participant fatigue. Survey versions were counterbalanced across participants; for attractiveness evaluations, 23 participants received version 1 and 22 received version 2, and for status evaluations, 20 received version 1 and 26 received version 2. Speech sample presentation order was randomized within survey versions.

### Procedure

After providing informed consent through the first section of the survey, participants completed demographic questions and questions on inclusion/exclusion criteria. Following this, they were instructed to ensure that they were in a quiet space with high-speed internet and computer volume set at an appropriate level. Participants were encouraged to close any open tabs/windows and use high quality earphones and were reminded to complete the survey in one sitting. Participants first completed a trial session with a sample from outside the stimulus set. During this trial, participants were informed that sliders on the scales may be placed between major divisions occurring every 10 units, and that they could move on to the next speech sample after a minimum of 6 s, if they chose to do so. Participants rated each stimulus on two 100-point, slider scales anchored at 1 “strongly disagree” and 100 “strongly agree.” For short-term and long-term attractiveness ratings, female participants indicated their agreement with the statements, *“This man is attractive for a short-term, purely sexual relationship”*, and “*This man is attractive for a long-term committed relationship”.* For dominance and prestige ratings, male participants indicated their agreement with the statements, *If this man got in a fistfight with an average male undergraduate, this man would probably win”*, and *“This man is a prestigious person who is respected, admired, talented, and successful”*. The order of statements was counterbalanced across participants within each of the two versions of both surveys. Following the single presentation of a speech sample, the scales appeared for 15 s before the next sample was presented. After evaluating speech stimuli for attractiveness or social status, participants evaluated the same 40 samples (presented in a different random order) for their agreement with the statement, *“This man sounds like someone who grew up in the same community as I did”.*

## Results

### Measures

For the first set of analyses (mixed ANOVA, ANCOVA, correlation), the ratings of short-term and long-term attractiveness by each of the 45 female participants for each of the 20 aspirated /t/ samples and the 20 unreleased /t/ samples were averaged. This yielded four sets of averaged ratings for every female participant: short-term aspirated /t/, long-term aspirated /t/, short-term unreleased /t/, and long-term unreleased /t/. In the same way, we obtained four sets of averaged ratings for each of the 46 male listeners: unreleased /t/ prestige, unreleased /t/ physical dominance, aspirated /t/ prestige, and aspirated /t/ physical dominance. Tests of normality using both Kolmogorov–Smirnov and Shapiro–Wilk tests showed that the four kinds of ratings for the 80 speech samples were normally distributed (all p > 0.5). Power analysis with G*power 3.1 for a mixed ANOVA revealed that for a small effect size with partial eta square of 0.04, α = 0.05, and power = 0.80, the estimated minimum sample size is 36.

### Attractiveness evaluations

A 2-way mixed ANOVA was conducted with mating context (short- vs. long-term) and /t/ articulation (aspirated vs. unreleased) as within-subjects factors. Survey version (1 or 2) was entered as a between-subject factor. There was no main effect of mating context (short- vs. long-term), *F*(1, 43) = 1.94, *p* = 0.170, or main effect of /t/ articulation (aspirated vs. unreleased), *F*(1, 43) = 0.20, *p* = 0.656. There was, however, a significant interaction between mating context and /t/ articulation, *F*(1, 43) = 11.01, *p* = 0.002, ${\eta }_{p}^{2}=$0.204. There was no significant interaction with survey version (*F* (1, 43) = 0.38, *p* = 0.539).

**Figure 1:**
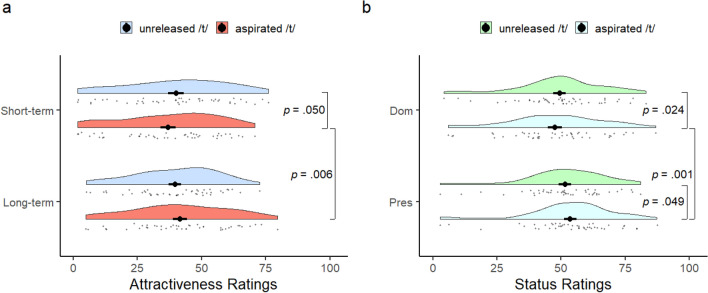
Differences in attractiveness and status ratings between unreleased /t/ versus aspirated /t/. Raincloud plots show (**a**) short-term and long-term attractiveness ratings, and (**b**) dominance and prestige ratings for unreleased /t/ versus aspirated /t/. The solid dot with lines extendending at the center of each of the rainclouds depicts means and standard errors. *Short-term* short-term attractiveness, *long-term* long-term attractiveness, *Dom* dominance, *Pres* prestige.

In line with hypothesis 2a, pairwise comparisons with Bonferroni corrections revealed a difference between long-term (*M* = 41.61, *SD* = 19.03) and short-term attractiveness scores (*M* = 36.95, *SD* = 19.28) for the aspirated /t/, *F*(1, 43) = 8.38, *p* = 0.006, ${\eta }_{p}^{2}=$0.163, 95% CI = [1.71, 7.78] with higher long-term scores. Confidence intervals, with 1000 bootstrap samples, for mean differences and correlations were calculated using MKinfer and bootcorci packages in R. Contrary to hypothesis 1a, a significant difference did not emerge between these scores for the unreleased /t/, *F*(1, 43) = 0.064, *p* = 0.802, 95% CI [− 3.93, 2.84] (see Fig. 1a). Supporting hypotheses 1b and against hypothesis 2b, while there was a significant difference in short-term attractiveness (unreleased /t/ *M* = 40.07, *SD* = 20.21; aspirated /t/ *M* = 36.95, *SD* = 19.28) between the two kinds of articulation *F*(1, 43) = 4.061, *p* = 0.050*,* ${\eta }_{p}^{2}= .086,$ 95% CI [− 6.32, − 0.14], these did not differ in long-term attractiveness *F*(1, 43) = 2.102, *p* = 0.154, 95% CI [− 0.67, 4.65] (see Fig. 1a).

When “highly similar” and “less similar” categories were entered as between-subject factors in the repeated measures analysis, in addition to the significant two-way interaction between mating context and /t/ articulation, *F*(1, 41) = 7.75, *p* = 0.008, ${\eta }_{p}^{2}= .159,$ there emerged a significant three-way interaction among mating context, /t/ articulation, and similarity to unreleased /t/ speakers,* F*(1, 41) = 5.58, *p* = 0.023, ${\eta }_{p}^{2}= .120,$ but no significant interaction among mating context, /t/ articulation, and similarity to aspirated /t/ speakers, *F*(1, 41) = 3.18, *p* = 0.082. Independent sample *t*-tests between the “highly similar” and “less similar” groups revealed significant differences in both short-term and long-term attractiveness scores for both unreleased and aspirated /t/ articulations, with the highly similar groups receiving higher ratings than the less similar groups (*p* < 0.05 for all).

To control for potential effects of *f*_o_ and formants (see Table 1 for descriptive statistics), we used speakers as the units of analysis in an ANCOVA, with attractiveness evaluations as the within-subjects factor, /t/ articulation as the between-subjects factor, mean *f*_o_ and formant position for each male speaker entered as covariates. The interaction between mating context and /t/ articulation remained significant, *F*(1, 76) = 11.61, *p* = 0.001, ${\eta }_{p}^{2}= 0.133$. There was no significant interaction with *f*_o_ (or mean centered *f*_0_) or with formant position on attractiveness-evaluations.

**Table 1:** Descriptive statistics of acoustic parameters.

Acoustic parameters	M	SD
F_0_	110.00	14.74
F1	492.40	37.53
F2	1450.22	53.27
F3	2461.26	88.81
Jitter	0.023	0.005

*F*_*0*_ is fundamental frequency, *F1, F2, and F3* are the first three formant frequencies*. N* = 80.

Correlation analyses revealed a significant negative correlation between short-term attractiveness and *f*_o_, (*r*(78) =  − 0.29, *p* = 0.009, 95% CI [− 0.49, − 0.07]), but not between formant position and* f*_o_, (*r* = − 0.14, *p* = 0.211, 95% CI [− 0.35, 0.09])_._ Long-term attractiveness did not correlate with formant position or fundamental frequency (p > 0.05).

### Status evaluations

A 2-way Mixed ANOVA was conducted with status evaluations (physical dominance and prestige) and /t/ articulation (unreleased and aspirated) as the two within-subject factors. The survey version (1 or 2) was entered as a between-subjects factor. There was a main effect of status evaluations (physical dominance vs. prestige) on the ratings, *F*(1, 44) = 8.65, *p* = 0.005, ${\eta }_{p}^{2}=0.164$, but there was no main effect of /t/ articulation (unreleased vs. aspirated), *F*(1, 44) = 0.00, *p* = 0.997. There was also a significant interaction between status evaluations and /t/ articulation, *F* (1, 44) = 7.66, *p* = 0.008, ${\eta }_{p}^{2}=0.148$. There was no significant interaction with the survey version (*F* (1, 43) = 0.99, *p* = 0.324).

Pairwise comparisons with Bonferroni corrections revealed several significant differences. For the aspirated /t/ samples, supporting hypothesis 4a, there was a significant difference between the physical dominance scores (*M* = 47.51, *SD* = 18.46) and prestige scores (*M* = 53.58, *SD* = 16.77), *F*(1, 44) = 12.39, *p* = 0.001, ${\eta }_{p}^{2}=0.22$, 95% CI [− 9.32, − 3.18]), with higher prestige scores; against hypothesis 3a, no such difference emerged for the unreleased /t/ samples, *F*(1, 44) = 2.55, *p* = 0.118, 95% CI [− 4.85, 0.42] (see Fig. 1b). Also, in line with our hypotheses 3b and 4b, there was a significant difference between the unreleased and aspirated /t/ articulation for physical dominance evaluations, *F*(1, 44) = 5.47, *p* = 0.024, ${\eta }_{p}^{2}=0.11$, 95% CI [− 3.50, − 0.23], and prestige evaluations, *F*(1, 44) = 4.10, *p* = 0.049, ${\eta }_{p}^{2}= .09$, 95% CI = [0.38, 3.85], with higher physical dominance scores for the unreleased /t/ (*M* = 49.43, *SD* = 16.80; aspirated /t/ *M* = 47.51, *SD* = 18.46) and higher prestige scores for the aspirated /t/ (*M* = 53.58, *SD* = 16.77; unreleased /t/ *M* = 51.51, *SD* = 16.09; see Fig. 1b).

When the highly similar and less similar categories were entered as between-subject factors in the repeated measures analysis, unlike attractiveness-evaluations, there were no significant three-way interactions among status evaluations, /t/ articulation, and the similarity categories; the two-way interaction between status evaluations and /t/articulation was not significant, *F* (1, 42) = 3.24, *p* = 0.079.

To control for potential effects of *f*_o_ and formants (see Table 1 for descriptive statistics), we used speakers as the units of analysis in an ANCOVA, with status evaluations as the within-subject factor, /t/ articulation as the between-subject factor, and mean *f*_o_ and formant position (standardized formant value, see Puts et al.^29^ for formant position calculation) for each male speaker entered as covariates. There was a significant interaction between status evaluations and /t/ articulation, *F* (1, 76) = 6.30, *p* = 0.014, ${\eta }_{p}^{2}=0.077$. Further, there was a significant interaction effect between status evaluations and mean *f*_o_ (*F* (1, 76) = 3.99, *p* = 0.049, ${\eta }_{p}^{2}=0.050$) and status evaluations and formant position (*F* (1, 76) = 6.12, *p* = 0.016, ${\eta }_{p}^{2}=0.074$). Adding mean-centered f_0_ as covariates yielded similar results.

Correlation analyses revealed a significant negative correlation between physical dominance and formant position (*r*(78) =  − 0.29, *p* = 0.009, 95% CI [− 0.48, − 0.07]), but not with *f*_o_, although it did show a negative association (*r* = − 0.19, *p* = 0.095, 95% CI [− 0.42, 0.02])_._ Prestige evaluations did not correlate with *f*_o_ or formant position (*p* > 0.05). Additional correlation analyses were conducted to compare ratings between attractiveness and status evaluations. The overall (combining aspirated and unreleased /t/ speakers) prestige ratings by men significantly correlated with the overall long-term attractiveness ratings by women (*r* = 0.307, *p* = 0.006, 95% CI  [0.12, 0.53], but not short-term attractiveness ratings, *r* = 0.11, *p* = 0.320, 95% CI [− 0.09, 0.35), and the overall physical dominance ratings correlated with short-term attractiveness (*r* = 0.31, *p* = 0.006, 95% CI [0.06, 0.51], but not long-term attractiveness, *r* = 0.06, *p* = 0.608, 95% CI [− 0.13, 0.29]).

Lastly, between the two groups of aspirated and unreleased /t/ speakers, the difference in formant position (*t*(78) = 0.24, *p* = 0.811, 95% CI [− 5.06, 7.96]) or the *f*_o_ (*t*(78) = 0.38, *p* = 0.703, 95% CI [− 0.31, 0.22]) was not significant. However, there was a significant difference in the jitter values of the two kinds of speakers (*t*(78) = 2.75, *p* = 0.004, 95% CI [0.001, 0.005]) with higher jitter for the aspirated /t/ (*M* = 0.024, *SD* = 0.004) than the unreleased /t/(*M* = 0.022, *SD* = 0.004).

### Additional analyses

To consider both stimuli and participants as random factors, we ran a linear mixed-model analysis using lme4, lmerTest, and emmeans (post-hoc) packages in R. Similar to earlier analyses, there were significant interaction effects between mating context and /t/ articulation (*β* = 5.1, SE = 1.44, *t* = 3.55, *p* < 0.001) and status and /t/ articulation (*β* = 4.00, *SE* = 1.17, *t* = − 3.42, *p* < 0.001).

Pairwise comparisons revealed similar results to previous analyses in that while hypotheses 1b and 2b were not supported, hypothesis 2a was supported, i.e., the aspirated /t/ speakers received higher long-term attractiveness scores than short-term attractiveness scores (*β* = 4.66, *SE* = 1.01, *z* = 4.59,* p* < 0.001). Unlike the previous analysis, however, 1a was not supported; short-term attractiveness was not different between aspirated and unreleased /t/ speakers. Pairwise comparisons for status and /t/ articulation were also similar to earlier analyses in that while we did not find support for hypothesis 4a, hypothesis 3a was supported; the aspirated /t/ speakers received higher prestige scores than physical dominance scores (*β* = 6.06, *SE* = 0.83, z = − 7.34, p < 0.001). Unlike earlier analyses, we failed to find support for hypotheses 3b and 4b; the aspirated /t/ speakers and unreleased /t/ speakers did not significantly differ in either physical dominance or prestige ratings.

Two additional findings that help to integrate the status results from the two statistical models, as discussed in the next section, are the following. The prestige scores received by the unreleased /t/ speakers were significantly higher than the physical dominance scores received by the aspirated /t/ speakers (*β* = 3.97, *SE* = 1.01, *z* = 3.93, *p* = 0.001). Also, the prestige scores received by the aspirated /t/ speakers were significantly higher than the physical dominance scores received by the unreleased /t/ speakers (*β* = 4.15, *SE* = 1.01, *z* = 4.11,* p* = 0.000).

## Discussion

Aspirated /t/ speakers were significantly more attractive to women in the long-term than in the short-term and were perceived by men to be significantly more prestigious than physically dominant. Women did not perceive unreleased /t/ speakers as significantly more attractive for the short-term than for the long-term; men did not perceive the unreleased /t/ speakers as more physically dominant than prestigious. The two groups of speakers did not differ from each other in long-term attractiveness and differed in short-term attractiveness in the ANOVA analysis but not in the linear mixed model.

Even though the two groups of men did not differ in their prestige or physical dominance in the second analysis (unlike the first analysis), additional findings from this analysis are worth noting: prestige ratings of the unreleased /t/ speakers were significantly higher than the physical dominance ratings of the aspirated /t/ speakers (but not different from their own physical dominance scores), and prestige ratings of the aspirated /t/ speakers were significantly higher than the physical dominance ratings of the unreleased /t/ speakers (and those of their own). Considering these findings along with the findings from the first analysis, it is reasonable to posit that while the prestige of unreleased /t/ speakers may be related to their physical dominance, the higher prestige scores for aspirated /t/ speakers are attributable to factors beyond physical dominance.

### Physical dominance, status, and short-term mating attractiveness

Men who employed the unreleased /t/, on average, had higher concentrations of circulating testosterone, a gonadal steroid hormone that facilitates aggression in same-sex competition and measured prosocial displays^91,92^. Even though the physical dominance ratings of the two groups of male speakers, as rated by other men, did not differ (in the second analysis), these men could have differed in threat potential in that the unreleased /t/ speakers may have been associated with a greater readiness to inflict costs, if need be (asymmetric contests)^93,94^. Because humans form coalitions, the ability to inflict costs on those that may act against the common interests of the group is socially valuable and worthy of deference^82,95–98^. This interpretation not only accords with the different testosterone levels in the two groups (and the findings from the first analysis which showed a significant difference in physical dominance ratings between the two groups), but also with the finding from the second analysis that the unreleased /t/ speakers’ prestige ratings, while not different from their own physical dominance ratings, were higher than the aspirated /t/ speakers’ physical dominance scores. Considering women’s evaluations of attractiveness at this juncture will be helpful.

Whether the unreleased /t/ speakers differed from the aspirated /t/ speakers in short-term attractiveness is inconclusive due to inconsistent findings from the two statistical tests. The dual mating hypothesis posits that women’s short-term and long-term preferences are geared towards seeking good genes and resources, respectively. According to this proposal, a low vocal pitch and other testosterone-mediated masculine traits are putative good gene indicators, and women’s preference for these indicators typically increase in the short term (reviewed in Thornhill and Gangestad^99^). The two groups of speakers did not differ in their fundamental frequencies which is consistent with the lack of consistent difference in short-term mating attractiveness between these groups. It is possible that testosterone differences between the two groups, likely cued by the varied /t/ articulation, did not reliably influence their short-term attractiveness. According to a version of the handicap hypothesis^100^ only males in good condition can allocate enough energy to develop and display testosterone-mediated traits while also keeping themselves healthy making these traits costly (and therefore) honest signals of mate quality^101,102^. With unreliable difference in short-term attractiveness between the two groups, it is unclear if preferences exhibited during short-term mating considerations are exclusively determined by male condition or quality cued by differences in testosterone. It may also be the case that increasing the external validity of contexts in which speech is elicited may more actively influence these evaluations than when these are sought for context-neutral, read speech (addressed in the next section). An alternative, but not mutually exclusive, proposal points to the possibility of women engaging in short-term liaisons as a mate-switching strategy helping them secure new long-term mates for a wider range of benefits than those involving a limited set of good gene traits^103^. Based on the mate-switching proposal it makes sense that the short-term attractiveness of the two kinds of speakers were not definitively different, as these evaluations are informed by self-perceived mate value/mate value discrepancies in terms of several cost–benefit tradeoffs.

### Prestige, status, and long-term mating attractiveness

The aspirated /t/ speakers’ prestige scores were higher than those of the unreleased /t/ speakers in the first analysis; in the second analysis, their prestige scores were higher than their own physical dominance scores (as in the first analysis) *and* those of the unreleased /t/ speakers. Apart from cost infliction ability, two factors that have been found to be strongly associated with high status are benefit generation ability and willingness^81,82,95,98,104^. Women’s comparable long-term attractiveness ratings for the two kinds of male speakers and their higher long-term attractiveness ratings for the aspirated /t/ speakers (compared to their short-term attractiveness ratings) suggest that women are privy to the two routes to men’s status^81,105^: the aspirated /t/ speakers’ path is perceived as prestige linked (possibly via benefit generation ability and willingness) and the unreleased /t/ speakers’ path to status is potentially perceived as one associated with cost inflicting potential. Further support for this dual route perspective comes from game theoretical models, which have shown that alternative strategies (e.g., hawk vs. dove strategies in animal fighting or fast vs. coy female mating strategies) can evolve and stabilize over evolutionary time (e.g., Maynard Smith’s evolutionarily stable strategies^106,107^).

Prestige is a phylogenetically younger strategy than physical dominance that presupposes a cognitive architecture supportive of imitative social learning and conformist transmission^108^. Consider, for example, that transitioning to farming from hunting and gathering may have facilitated the emergence of the labiodental speech sounds /f/ and /v/. Farming resulted in softer diet which led to the preservation of the adolescent bite configuration into adulthood supporting the birth of these novel speech sounds^109^. The sociolinguistic implication is that the labiodental sounds could have been markers of high socioeconomic status possibly by virtue of their association with novel food production methods, prodigious output, and relative sedentism; thus, their adoption into languages may have resulted from prestige-driven processes^109^. The question that logically follows is what potential benefits or skills may be uniquely associated with the aspirated /t/ speakers such that prestige is freely and *distinctly* conferred upon them.

The aspirated /t/ has higher sociolinguistic status for reasons that may involve enhanced articulatory clarity. Given that prestigious individuals are models whom others perceive to be equipped with potential fitness-enhancing information/skill, it is reasonable to ascribe the role of a high-fidelity information carrier to articulatory clarity,^65,110^ a significant point considering the primacy of oral traditions before the advent of writing systems. Following this line of logic, articulatory behavior can understandably gain traction over time as one worthy of substantial focus and training. This conjecture also overlaps with the sociolinguistic literature on standard and non-standard differentiations of spoken language where the former, characterized by its focus on phonetic distinctiveness among other things, is promoted in formal situations. Given that accents crystallize by puberty^111^, it is reasonable to suggest that individuals uttering the aspirated /t/ at the end of words may be perceived as having experienced an early social history associated with high socioeconomic status and prestige. As women place a high importance on socioeconomic status, especially in long-term relationships, and men are in competition with other men for access to resources, attention to the prestige marker by both sexes is not surprising. When perceived similarity to speakers was high, this also increased speakers’ attractiveness scores supporting the effect of familiarity in speech-based social perceptions^12,112,113^ and the possibility of assortative mating. The association of /t/ production with testosterone, however, sheds doubt on a purely sociocultural explanation (although testosterone levels are also regulated by the social environment^114,115^).

The higher the testosterone, the lower the likelihood of aspirated /t/ production, and vice versa^62^. Yet, neither the formant position nor the *f*_*0*_ of the two groups of men were significantly different. There, however, emerged a difference in the jitter values of the two kinds of speakers with the aspirated /t/ articulation demonstrating higher jitter. If the men differed in their vocal-verbal strategy, i.e., recurrent shifts between laryngeal lowering and raising vs. relatively constant lowering, it is reasonable to expect changes in jitter, as it tracks local variations in *f*_o_ resulting from prosodic variations^116^. Note that the neural basis for laryngeal rather than supralaryngeal manipulation is unique to humans among primates^117–120^, which further highlights the potential for variability in this behavior. It is also possible that if some of these individuals grew up in a linguistic environment favoring the aspirated /t/, this early experience would be manifested in their speech, as active articulatory manipulations can overcome constraints posed by laryngeal height^121^ or other oral structural changes^122^. Note that the study used only the extremes of a broad range of samples; the unused middle of the spectrum had many samples comprising different proportions of unreleased and aspirated /t/s. Individual differences in strategy adoption mediated by testosterone levels in combination with exposure to specific linguistic environments may determine the frequency of use of these allophones, which needs to be empirically verified in the future.

Consider the trade-offs that potentially render /t/ productions as part of a costly signal. Enhancement of one aspect supporting a multifaceted trait such as spoken language may compromise other aspects. For example, extraordinarily high levels of circulating testosterone and fetal testosterone have been shown to reduce fluency^123^ and vocabulary size^124^ (see Whitehouse et al.^125^ for association between high prenatal testosterone levels and language delay). Activational effects of testosterone in adults have also been linked with similar trade-offs between cognitive and verbal aspects^126^ and the structure and connectivity of language-specific brain regions^127^. Based on the trade-off perspective, men who exhibit articulatory clarity *and* masculinity via vocal pitch (and other morphological indicators) may be signaling a costly handicap, thereby a costly signal of a well-integrated individual. Competitive contexts epitomized by debates that require vocal-verbal displays of assertiveness, nimbleness, and civility may be especially relevant in studying both status and mate choice-relevant evaluations, as these contexts likely reveal cost inflicting and benefit generating propensities of speakers. It is relevant to note here that men significantly outnumber women as participants and winners in extemporaneous debates^128,129^. And, in one study, American boys in orthodox Jewish communities produced more aspirated /t/’s than girls did, especially when debating^11,14^. These sex-differentiated findings with respect to the likelihood of engaging in competitive verbal performances and the verbal clarity exhibited in these events highlight the need to examine vocal-verbal displays in evolutionarily relevant contexts.

Articulation accuracy in early development is predictive of intelligence^130^ and executive function^131^, findings that support the association of clear enunciation and high prestige. Also, articulatory precision is affected by neurocognitive and neuromotor disorders^12^. This suggests that the capacity to imitate sounds may indicate a healthy cognitive-motor apparatus and/or the likelihood of conforming to prescribed or expected standards (e.g., see Yu et al.^132^ for individual differences in the extent of phonetic accommodation to model talkers; also, Pardo et al.^133^). In some cultures, behavioral conformity is associated with intelligence, and in others, non-conformity is valued (see discussion in Durkee et al.^95^). If the aspirated /t/ speakers were deemed more attractive for the long-term than for the short-term it is also not unreasonable to attribute this finding to their potential non-availability for short-term flings. A substantial sexual history may not necessarily be reputation-enhancing for both sexes considering that our species is characterized by pair bonding, biparental care, and the capacity for linguistic-cultural transmission^134^, and relationship fidelity may be a moral virtue that may have been a potential candidate for mutual mate choice in humans^135^.

While low vocal pitch in men is a secondary sexual characteristic, most likely driven by sexual selection, less attention has been given to the *use* of low voice pitch as a behavioral strategy^136,137^. Considering vocal pitch flexibility as an integral aspect of the act of speaking guides us to a collection of potential cues/signals including interactions and possible interferences among vocal pitch, articulatory parameters, fluency indices, and linguistic components^27^, opening an interesting avenue of research. Speech in real life (unlike lab-based read speech) is a complex and costly signal (see Locke^27^) that is hard to fake and likely demonstrates a wide range of individual differences, and some individuals may be better verbal performers than others displaying context-specific vocal and verbal flexibility. Past research also indicates that women’s vocal^30^ and articulatory^12^ patterns are highly salient, and have important implications for attractiveness and status perceptions. Future studies should focus on this area as well.

## Conclusion

Using evolutionary principles built upon the Darwinian theoretical foundation, we wove together findings from several disciplines including speech science, phonetics, sociolinguistics, biology, anthropology, and psychology to generate novel hypotheses. Most of these were supported by our data. Nevertheless, our samples were restricted to WEIRD groups, i.e., western, educated, industrialized, rich, and democratic^138,139^, and the word-ending /t/ patterns and related evaluations may not apply to all English speakers, such as non-native speakers, or to speakers of other languages. We note, however, that word-final unreleased stops have been discussed as a proto-Indo-European feature evident even in distantly related daughter languages^140^. Even though specific sounds and sound combinations vary across languages, the distinctive feature of lenition (weakness) as opposed to tenseness in word-final sounds seems to be ubiquitous,^140^suggesting that countering the weakened finale with a more intense sound may exhibit attention to detail (that closely matches writing conventions) or a general state of arousal; this opens the door to new research hypotheses on plausible prestige markers cross-linguistically and potentially dichotomous social evaluations related to the two paths to status. Future work would benefit from multidisciplinary collaborations that include historical linguists and phoneticians. Moving deeper into the realm of speech articulation enables us to explore potential selection pressures hinging on our relatively unique capacity for linguistic-cultural transmission while also examining the influence of evolutionarily ancient traits.

## Data Availability

The data have been made available online at https://osf.io/hdap2/?view_only=00c0e24dfe484bf69f8b209815deb16e.
